# mTOR and ROS regulation by anethole on adipogenic differentiation in human mesenchymal stem cells

**DOI:** 10.1186/s12860-018-0163-2

**Published:** 2018-07-06

**Authors:** Yun-Hee Rhee, Jeong Hwan Moon, Ji-Hun Mo, Tiffany Pham, Phil-Sang Chung

**Affiliations:** 1 Beckman Laser Institute Korea, Dankook University, 119 Dandae-ro, Cheonan, 31116 Republic of Korea; 2 Laser Translational Clinical Trial Center, Dankook University Hospital, Cheonan, 31116 Republic of Korea; 3 Department of Otolaryngology-Head and Neck Surgery, College of Medicine, Dankook University, Cheonan, 31116 Republic of Korea; 4 Beckman Laser Institute and Medical Clinic, University of California, Irvine, 1002 Health Sciences Rd, Irvine, CA 92612 USA

**Keywords:** Adipogenesis, hMSC, Anethole, ROS, AMPK, mTOR

## Abstract

**Background:**

Adipocyte differentiation of human mesenchymal stem cells (hMSCs) is dependent on mitochondrial metabolism and reactive oxygen species (ROS) to initiate adipocyte differentiation. Although anethole has been known as an anti-oxidant and lipid peroxidation inhibitor, there is little investigated about its role in adipogenic differentiation.

**Methods:**

The effects on cytotoxicity and proliferation of anethole in hMSCs were measured by the MTT assay. The anti-adipogenic effect of anethole on hMSCs was analyzed by Oil Red O staining and western blot analysis. The anti-oxidant activity of anethole on hMSC was assessed by flowcytometry and fluorescence staining using 2',7' –dichlorofluorescin diacetate (DCFDA). The western blotting was used to detect of phospho-Akt, phospho-mTOR, phospho-p70S6K, PPARγ, and phsopho-AMP-activated kinase (AMPK).

**Results:**

Anethole suppressed the adipogenic differentiation of hMSCs through down-regulation of Akt-mTOR-p70S6K-PPARγ and up-regulation of AMPK. Anethole affected oxidative conditions through ROS generation. Anethole also rescued AMPK activity and reduced activation of mTOR-p70S6K-PPARγ under oxidative conditions in presence of exogenous hydrogen peroxide.

**Conclusion:**

ROS and mTOR regulation is a crucial factor in adipogenic differentiation, anethole has an important role in regulating activities of mTOR/PPARγ and ROS control in adipogenic differentiation of hMSCs.

**Electronic supplementary material:**

The online version of this article (10.1186/s12860-018-0163-2) contains supplementary material, which is available to authorized users.

## Background

Adipogenic differentiation of human mesenchymal stem cells (hMSCs) is characterized by mitochondrial metabolism [1, 2]. During adipogenic differentiation, autophosphorylation of insulin/insulin-like growth factor 1 (IGF-1) receptor tyrosine kinase in the presence of insulin initiates glucose transport, glucose metabolism, proadipogenic gene transcription and de novo lipid synthesis. In addition, Akt activation downstream of insulin signaling to produce the mammalian target of rapamycin (mTOR) complex further contributes to adipocyte differentiation [3–5]. Recent studies have revealed that the mTOR signaling pathway has a critical role in the regulation of adipose tissue function [6], including adipogenesis [7], and lipid metabolism [4, 5]. While exploring the differentiation pathway of mesenchymal stem cells, we found that mTOR has diverse functions, not only as an oncogene but also as an adipogenic inducer of peroxisome proliferater-activated receptor gamma (PPARγ) through p70S6 kinase (p70S6K). In our previous study, we demonstrated that anethole had an effect on mTOR suppression [8]. Furthermore, PPARγ was related to the dependent signaling of cyclic adenosine monophosphate (cAMP), whose synthesis was inhibited oxidative stress [9]. When hMSCs from bone marrow were exposed to adipogenic inducing media, hMSC displayed robust lipid accumulation within 21 days [3]. In this process, basal oxygen consumption rate was coupled to the generation of ATP synthesis and intracellular ROS was increased [10]. In this regard, ROS and mTOR regulation may play a crucial role in the differentiation of adipocytes in mesenchymal stem cells.

**Fig. 1:**
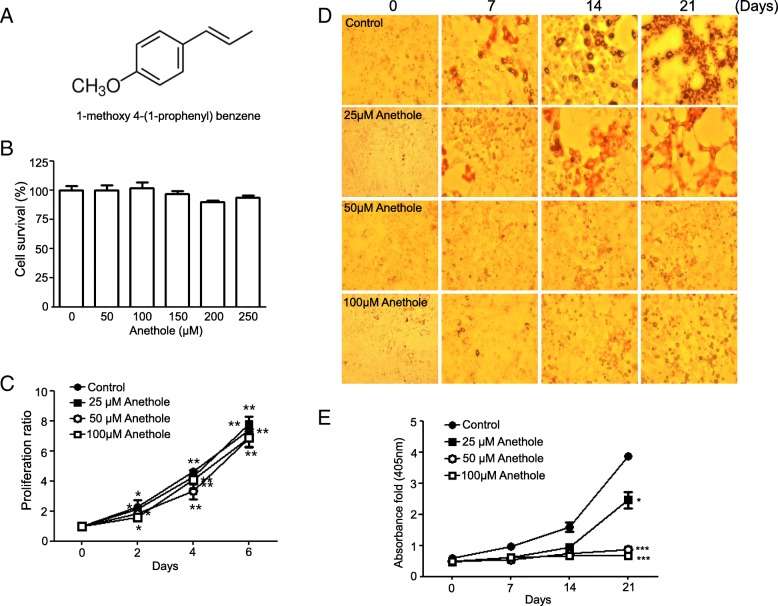
Effect of anethole on cytotoxicity and proliferation in hMSCs. **a** The structure of anethole (1-methoxy-4-(1-propenyl) benzene). **b** Cells were seeded onto a 96-well plate at a density of 5000 cell/well, and were treated at various concetrations (0, 50, 100, 150, 200, and 250 μM) for 24 h. The cell viability was calculated as a percentage of viable cells in anethole-treated group vesus the untreated control. **c** For the proliferation assay, every step was performed as described previously and assessment was made at days 2, 4, and 6 post anethole treatment. Each experiment was repeated three times and the values are presented as mean ± S.D. **d** hMSCs were incubated in adipogenic differentiation media for 4 weeks in presence or absense of 50 μM anethole. Cells were fixed with 4% formaldehyde and stained with 0.5% Oil Red O staining solution at first day of each week for 4 weeks and phostographed by microscopy. **e** The stained-lipid accumulation was measured by dissoloving the cell contents in isopropanol and reading their absorbace at 405 nm by a microplate reader. Each experiment was repeated four times and the values are presented as mean ± S.D. *p < 0.1, **p < 0.05, and ***p < 0.001

Anethole, or 1-methoxy-4-(1-propenyl) benzene, is an aromatic compound (Fig. 1a) that occurs widely in nature which has been reported to have an effect to various disease, such as inflammation [11, 12], cancer [8, 13], and toxicity [14, 15]. These biological activities are known to be attributed to anti-oxidant activity [16], lipid-peroxidation inhibition [17] and hydroxyl radical scavenging [8, 18]. These functions of anethole as an antioxidant depend on how ROS are efficiently regulated both inside and outside the cell. ROS generation is also required to initiate adipocyte differentiation and glycogen synthse kinase activation in glucose metabolism [9]. In this study, we hypothesized that anti-adipogenic effect of anethole is due to its anti-oxidant function and energy metabolism regulation through mTOR suppression. We examined whether adipogenic differentiation of hMSCs was affected by ROS and mTOR, which are regulated by anethole.

## Methods

### Cell culture and reagents

Human bone marrow derived mesenchymal stem cells (hMSCs, Cat# PT-2501) were obtained from Lonza (Walkersville, MD, USA) and cultured according to the manufacturer’s instructions. For adipocyte differentiation, cells were cultured for 4 weeks in high-glucose (25 mM) Dulbecco’s Modified Eagles Medium (DMEM) supplemented with 10% fetal bovine serum, 1% penicillin**-**streptomycin, 200 μM indomethacin, 1 μM dexamethasone, 10 μg insulin, and 0.5 mM isobutylmethylxanthine. DMEM, FBS, and penicillin-streptomycin were purchased from Corning (Oneonta, NY, USA). Components of adipogenic induction media and all reagents including anethole were purchased from Sigma-Aldrich (St. Louis, MO, USA).

### Cytotoxicity and proliferation assay

The MTT [3-(4,5-dimethylthiazol-2-yl)-2,5-diphenyltetraxolium bromide] tetrazolium reduction assay was performed to determine the cell viability and proliferation. For cytotoxicity assessment, the cells were seeded onto a 96-well plate at a density of 5000 cells per each well. The cells were treated with anethole at various concentrations (0, 50, 100, 150, 200 and 250 μM). After 24 h, 5 mg/ml MTT was added to each well and incubated until formazan was produced. Formazan was dissolved with the MTT lysis solution (20% SDS, 50% dimethylformamide). The plates were measured for optical density (OD) using a microplate reader (TECAN, Männedorf, Zürich, Switzerland) at an absorbance wavelength of 450 nm. Cell viability was calculated as a percentage of viable cells in the anethole-treated group versus the untreated control by the following equation:. Cell viability (%) = [OD (anethole) – OD (Blank) / OD (Control) – OD (Blank)] × 100. Every step was performed as previously described for proliferation assessment at days 2, 4, and 6 post anethole treatment. The proliferation ratio was calculated allowing for daily variation according to the cell viability equation. Each experiment was repeated three times.

### Oil red O staining and lipid accumulation assay

hMSCs were incubated in adipogenic differentiation media for 4 weeks in presence or absence of 50 μM anethole. Cells were then fixed with 4% paraformaldehyde and stained with 0.5% Oil Red O staining solution on the first day of each week and imaged using microscopy. After photographing the cells at 200× magnification, stained lipid accumulation was measured by dissolving the cell contents in isopropanol and reading their absorbance at 450 nm by a microplate reader (Biochrom, Cambridge, England).

### Flowcytometry analysis of ROS

For exogenous ROS study, hMSCs were exposed at 2 mM H_2_O_2_ for 30 min and incubated for 2 days in presence or absence of 50 μM anethole. ROS were measured by staining the cells with DCFDA cellular ROS detection assay kit (Abcam, Cambridge, MA, USA) according to the manufacturer’s protocol. After staining, cells were strained briefly and analyzed using Accuri-C6 (BD, Bedford, MA, USA). ROS generation was also observed under a fluorescence microscope (BX51, Olympus, Miami, FL, USA) and these samples subsequently underwent western blot analysis for p-mTOR and p-AMPK (Cell signaling, Beverly, MA, USA).

### Western blot analysis

hMSCs were pretreated with 50 μM anethole and incubated in adipogenic differentiation media for 3 weeks. The lysates were prepared with 100 μl of lysis buffer (50 mM Tris-HCl, pH 7.4, 300 mM NaCl, 0.5% Triton X-100, 5 mM EDTA, 1 mM Na_3_VO_4_, 1 mM NaF, 10 μg/ml aprotinin, 10 μg/ml leupeptin, 10 μg/ml pepstatin, 10 mM iodoacetamide, 1% phenylmethylsufonyl fluoride, PMSF) for 30 min on ice at every week point after treatment for 4 weeks. Total protein extracts at 30 μg were separated with SDS-PAGE and electro-transferred onto a Hybond ECL membrane with the transfer buffer (25 mM Tris, 250 mM glycine, 10% methanol). The membranes were blocked with 5% BSA in TBST and immunoblotted for phospho-mTOR (Ser 2448), mTOR (Cell signaling), phospho-70S6K (ThermoFisher, Waltham, MA, USA), PPARγ (Cell signaling), phospho-Akt, Akt, phospho-AMPK, AMPK (Cell signaling) and β-actin (Sigma, St. Louis, MO, USA). After washing with TBST, the membranes were incubated with HRP-conjugated secondary antibody and developed using an ECL detection kit (GE Healthcare, Pittsburgh, PA, USA). Each protein expression was normalized by β-actin and calculated using the image J program (https://imagej.nih.gov/ij/). To study exogenous ROS, 2 mM H_2_O_2_ was added for 30 min at first day of adipogenic differentiation. After exposure to H_2_O_2_, hMSCs were incubated with adipogenic media for 2 weeks. Whole cell lysates were prepared equally at day 14 as described previously.

### Statistical analysis

All data were expressed as mean ± standard deviation (S.D.). The differences between the treatment groups and untreated controls were calculated by the Student’s *t*-test one way ANOVA (Tukey test) using Prism (GraphPad, La Jolla, CA, USA). Statistical significance was determined at a value of *p* * < 0.1, p** < 0.05, and p*** < 0.001.

## Results

### Effect of anethole on cytotoxicity and proliferation of hMSCs

Anethole showed no cytotoxicity towards hMSCs up to a concentration of 250 μM. The cell survival rate for anethole was 100.37 ± 9.49% at 50 μM, 102.04 ± 11.71% at 100 μM, 96.78 ± 6.2% at 150 μM, 90.28 ± 3% at 200 μM, and 93.6 ± 4.88% at 250 μM. The mean survival rate at 200 and 250 μM was slightly decreased, but not significantly (Fig. 1b). Anethole did not affect cell proliferation until it reached a concentration of 100 μM. As shown in Fig. 1c, the cell proliferation was similar in control and anethole-treated groups after 6 days.

### Effect of anethole on adipogenic differentiation in hMSCs

**Fig. 2:**
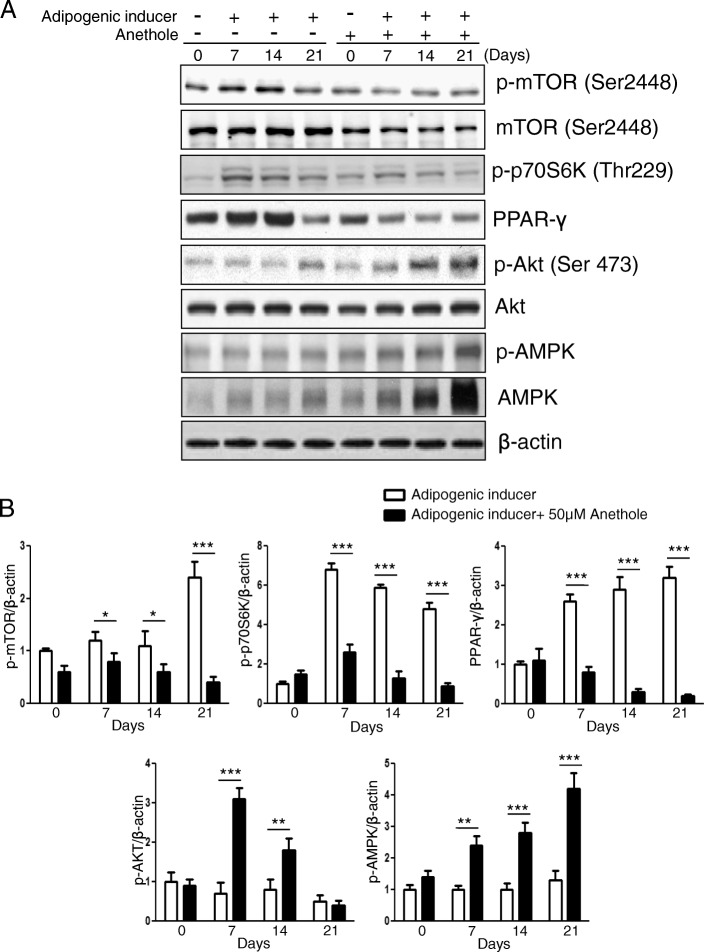
Western blot analysis of anethole on adipogenic differentiation in hMSCs. **a** hMSCs were pretreated with 50 μM anethole and incubated in adipogenic media for 4 weeks. Once a week, whole cell pellets were lysed in lysis buffer and immnoblotted for p-mTOR, mTOR, p-p70S6K, p70S6K, PPAR-γ, p-Akt, Akt, p-AMPKand AMPK. **b** The phosphorylated expression of each protein was caculated by dividing the total form of corresponding protein, and was normalized by β-actin. The relative folds were measured and calculated using Image J. Each experiment was repeated three times and the values are presented as mean ± S.D. **p < 0.05, and ***p < 0.001

We followed the adipogenic differentiation of hMSCs by Oil Red O staining. As the adipogenic differentiation progressed by the adipogenic inducer, the lipid accumulation was increased and with the displays of red after Oil Red O staining. As shown in Fig. 2a, hMSCs had robust lipid accumulation within 3 weeks when exposed to the adipogenic inducer cocktail of indomethacin, dexamethasone isobutylmethylxanthine (IBMX), and insulin. However, lipid accumulation was diminished (25 μM anethole) or removed (50 and 100 μM anethole) after treatment with anethole. The microscope observations are shown in Fig. 1d. The quantification of lipid accumulation by measuring absorbance was verified Fig. 1e.

### Western blot analysis of anethole on adipogenic differentiation in hMSCs

To investigate if adipogenic differentiation inhibition on hMSCs by anethole was dependent on the mTOR-PPARγ axis and AMPK, we performed a western blot analysis. As shown in Fig. 2, the phosphorylation of mTOR, p70S6K, and PPARγ rapidly increased under the induction of adipocyte differentiation and remained until 3 weeks. The phosphorylation of AMPK was reduced or unchanged with adipogenic induction of hMSCs. However, the adipogenic differentiation through activation of mTOR-p70S6K-PPARγ was completely inhibited by anethole treatment. The phosphorylation of mTOR decreased from the first week, whereas p70S6K and PPARγ started to decrease from the second week by anethole, and remained low. Conversely, AMPK phosphorylation increased after anethole treatment, and remained high.

### Effect of anethole on ROS in hMSCs

**Fig. 3:**
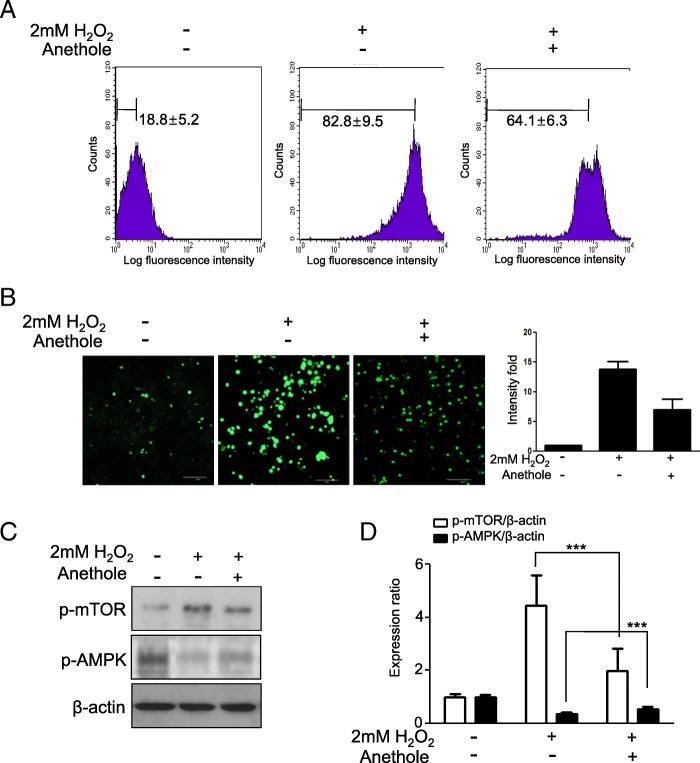
Effect of anethole on excessive ROS generation in hMSCs. **a** hMSCs were exposed at 2 mM H_2_O_2_ for 30 min and incubated for 2 days in presence or absence of 50 μM anethole. ROS was measured by staining the cells with DCFDA cellular ROS detection assay kit according to the manufacturer’s instructions. After staining, cells were strained briefly and analyzed using Accuri-C6. **b** ROS generation was also observed under a fluorescence microscope at 200× magnification after same treatment previously described. **c** Whole cell pellets underwent western blot analysis for p-mTOR and p-AMPK. **d** Each protein expression was exhibited in the same manner as described in Fig. 3b. Each experiment was repeated three times and the values are presented as mean ± S.D. **p < 0.05, and ***p < 0.001

We hypothesized that ROS has a critical role on adipogenic differentiation of hMSCs. We assessed whether anethole has an effect on transcriptional programming via mTOR and AMPK by reducing excessive ROS. Hydrogen peroxide is the major form of ROS that triggers redox dependent signaling in the cytosol [10]. hMSCs were exposed at 2 mM H_2_O_2_ for 30 min and incubated for 2 days in the presence or absence of 50 μM anethole, and subsequently stained with DCFDA. After staining, cells were analyzed by flow cytometry and fluorescence microscopy. As shown in Fig. 3a and b, ROS in hMSCs was present in some degree (18%) in the initial stage, increasing to about 82% after the treatment with hydrogen peroxide and decreasing to about 64% in the presence of anethole. We also investigated the expression of mTOR and AMPK under oxidative stress with or without anethole by western blot analysis. As shown in Fig. 3c, ROS induced phosphorylation of mTOR and de-phosphorylation AMPK, which effects were diminished by anethole.

### Effect of anethole on the adipogenic markers under excess ROS in hMSCs

**Fig. 4:**
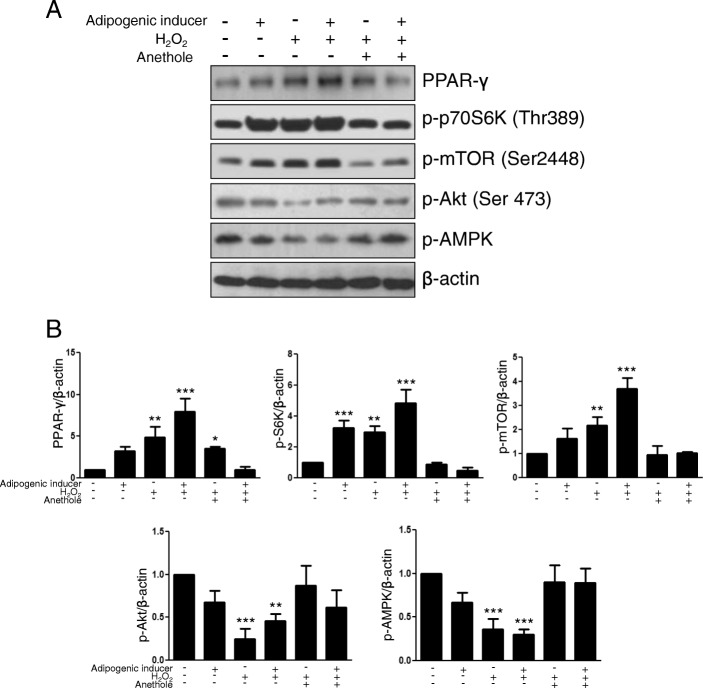
Western blot analysis of theffect of anethole on transcriptional factors with excessive ROS during adipogenic differentiation. **a** 2 mM H_2_O_2_ was added for 30 min on the first day of adipogenic differentiation. After exposure to H_2_O_2_, hMSCs were incubated with adipogenic media for 2 weeks. Whole cell lysates were prepared equally at day 14 as described in Fig. 3. **b** Each protein expression was exhibited in the same manner as described in Fig. 3b. Each experiment was repeated three times and the values are presented as mean ± S.D. **p < 0.05, and ***p < 0.001

To examine whether the increase in ROS affects adipogenic differentiation, hMSCs were exposed to ROS and cultured for expression of PPARγ, a terminal adipogenic marker. As shown in Fig. 4, H_2_O_2_ significantly upregulated the expression of PPARγ and the mTOR-p70S6K signal axis and decreased expression of AMPK. However, the PPARγ/ mTOR-p70S6K signal axis was significantly decreased in the presence of anethole, and the expression of AMPK was also restored by anethole despite the presence of H_2_O_2_.

## Discussion

Adipogenic differentiation is a developmental process that is critical for metabolic homeostasis and nutrient signaling. mTOR kinase mediates nutrient signaling to regulate cell growth [19], proliferation [5], and diverse cellular differentiation pathways [5, 20]. In addition, AMPK is known as a major regulator of cellular energy homeostasis and is involved in various metabolic pathways [21]. During adipogenic differentiation, AMPK and mTOR play unique roles. mTOR facilitates the accumulation of triglycerides by promoting adipogenesis and lipogenesis and by shutting down catabolic processes such as lipolysis and β-oxidation. Meanwhile, AMPK activation leads to energy preservation for cell survival at the expense of growth and proliferation via long term transcriptional control of key players of various metabolic pathways.

In this study, we assessed whether anethole could regulate mTOR and AMPK activation and could abolish excess ROS from oxidative stress during adipogenic differentiation in hMSCs. We evaluated the change of Akt-mTOR-PPARγ axis and AMPK phohsphorylation under oxidative stress in the presence of anethole. Although anethole has been known to not only be an anti-oxidant [16] but also a lipid-peroxidation inhibitor [17], little has been studied about its role in adipogenic differentiation. As shown in Fig. 1, cell viability and proliferation were not affected by anethole up to 100 μM. However, anethole inhibited the lipid accumulation of hMSCs under adipogenic inductive conditions without any changes in cytotoxicity. We investigated whether the inhibition effect on adipogenic differentiation of hMSC by anethole was dependent on the major adipogenic signaling cascade of Akt-mTOR-p70S6K-PPARγ. As we expected, anethole inhibited the phosphorylation of mTOR, p70S6K, PPARγ and Akt. Interestingly, the activation of AMPK was detected under presence of anethole during adipogenic differentiation of hMSCs (Fig. 2). mTOR has been reported as a major downstream effector in PI3K/Akt signaling [22], and AMPK had a contrasting activity to mTOR [2]. AMPK activation in the early phase of differentiation inhibits PPARγ and C/EBPs expression as well as late adipogenic markers such as fatty acid synthase (FAS) and acetyl-coA carboxylase. Several studies have revealed the convergence of AMPK and mTOR signaling pathways, pointing to mTOR as a central signal integrator that receives signals arising from growth factors, nutrients, and cellular energy metabolism [4, 23]. Mitochondrial biogenesis and metabolism are thought to be important for MSC differentiation and among them, it is presumed that the occurrence of ROS plays a crucial role in adipogenesis [24]. MSCs have low antioxidant activity and are more sensitive to oxidative stress compared to more differentiated cell types [10]. With regard to adipogenesis, ROS increases as MSCs differentiate into adipocytes, but it is unclear whether this is a cause or consequence of adipogenesis. Thus, we investigated whether excessive ROS from the addition of exogenous H_2_O_2_ induced the imbalance between mTOR and AMPK. We examined that H_2_O_2_ treatment induced excessive ROS in hMSCs by flow cytometry analysis and fluorescence microscopy observation using DCFDA (Fig. 3a and b). The phosphorylation of mTOR was elevated and the phosphorylation of AMPK was diminished after H_2_O_2_ treatment. However, anethole down-regulated the phosphorylation of mTOR, and up-regulated the phosphorylation of AMPK. These finding demonstrated that ROS affect the metabolic pathway between mTOR and AMPK, which could be regulated by anethole (Fig. 3c and d). Next, we assessed the expression of Akt-mTOR-PPARγ signaling during adipogenic differentiation of hMSCs with and without oxidative conditions. The phosphorylation of mTOR-p70S6K-PPARγ was accelerated only by the oxidative condition. These results demonstrated that ROS was required for activation of the transcriptional machinery for adipogenic differentiation. However, anethole regulated the over-expression of adipogenic markers and restored the AMPK activation. In particular, phosphorylation of Akt was decreased under oxidative condition without adipogenic induction, which suggests that it was relatively sensitive to oxidative stress of MSCs themselves (Fig. 4).

Several studies reported that antioxidant enzymes such as superoxide dismutase (SOD), catalase, and glutathione peroxidase (GPX) are upregulated during adipognesis in hMSCs [25], and that the ROS scavenger N-acetylcysteine (NAC) inhibits adipogenesis in the murine MSC cell line [26]. Our most significant finding indicated that ROS were crucial activators and that mTOR was a central signal integrator of cellular energy metabolism in hMSCs during adipogenic differentiation. Our results also suggested that anethole was not only an ROS scavenger but also an inhibitor of adipogenic pathways such as Akt-mTOR-PPARγ. Altogether, these findings suggest anethole may provide an anti-obesity signal by regulating cellular metabolism through ROS and mTOR.

## Conclusion

ROS and mTOR regulation is a crucial factor in adipogenic differentiation, and anethole has a potent to regulate the activities of mTOR/PPARγ and ROS in adipogenic differentiation of hMSCs.

## Additional files

Additional file 1: The raw data of western blot. PPAR-γ, p-Akt, and β-actin in Fig. 2. (JPG 120 kb)

Additional file 2: The raw data of western blot. Akt, mTOR and AMPK in Fig. 2. (JPG 113 kb)

Additional file 3: The raw data of western blot. p-mTOR, p-AMPK, and p-p70S6K in Fig. 2. (JPG 114 kb)

Additional file 4: The raw data of western blot. PPAR-γ, p-AMPK, and and β-actin in Fig. 4. (JPG 120 kb)

Additional file 5: The raw data of western blot. p70S6K, p-mTOR, and p-Akt in Fig. 4. (JPG 106 kb)
