# Supplementary figures and images for: mTOR and ROS regulation by anethole on adipogenic differentiation in human mesenchymal stem cells
Source: BMC Cell Biol. 2018 Jul 6;19:12. doi: 10.1186/s12860-018-0163-2 (PMC6035441; doi:10.1186/s12860-018-0163-2)

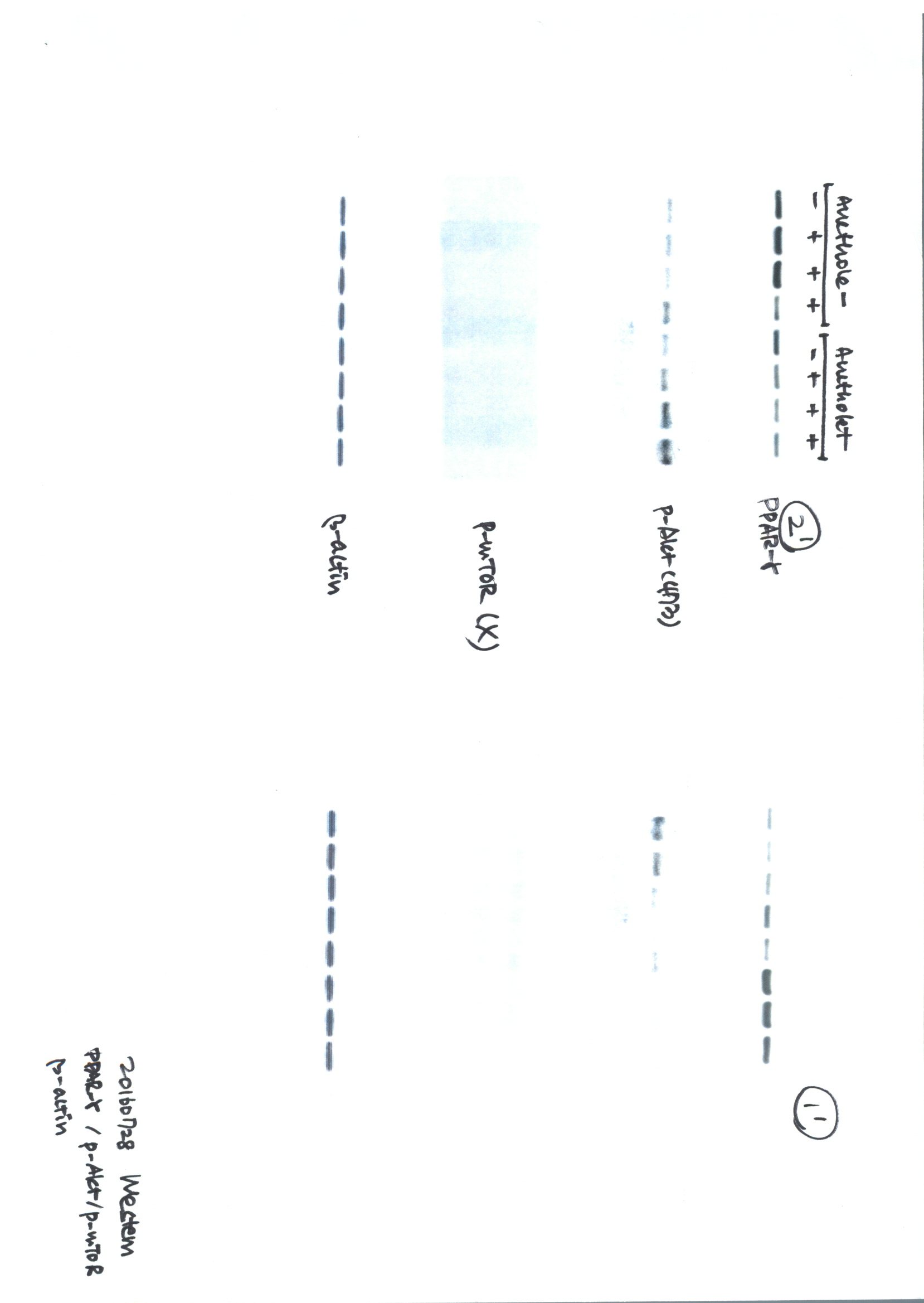

Supplement: Supplementary file 1 — The raw data of western blot. PPAR-γ, p-Akt, and β-actin in Fig. 2. (JPG 120 kb) [file 12860_2018_163_MOESM1_ESM.jpg]

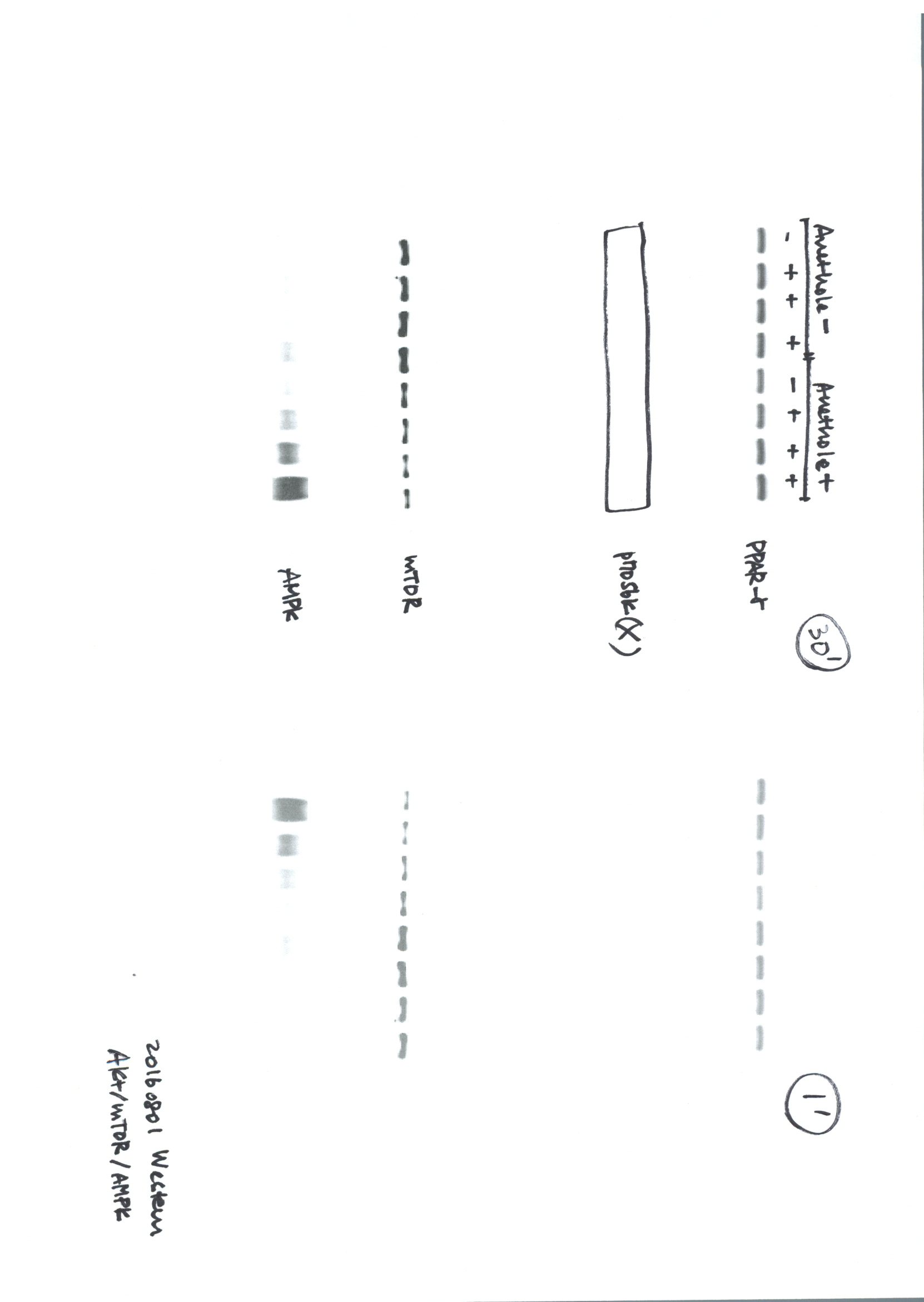

Supplement: Supplementary file 2 — The raw data of western blot. Akt, mTOR and AMPK in Fig. 2. (JPG 113 kb) [file 12860_2018_163_MOESM2_ESM.jpg]

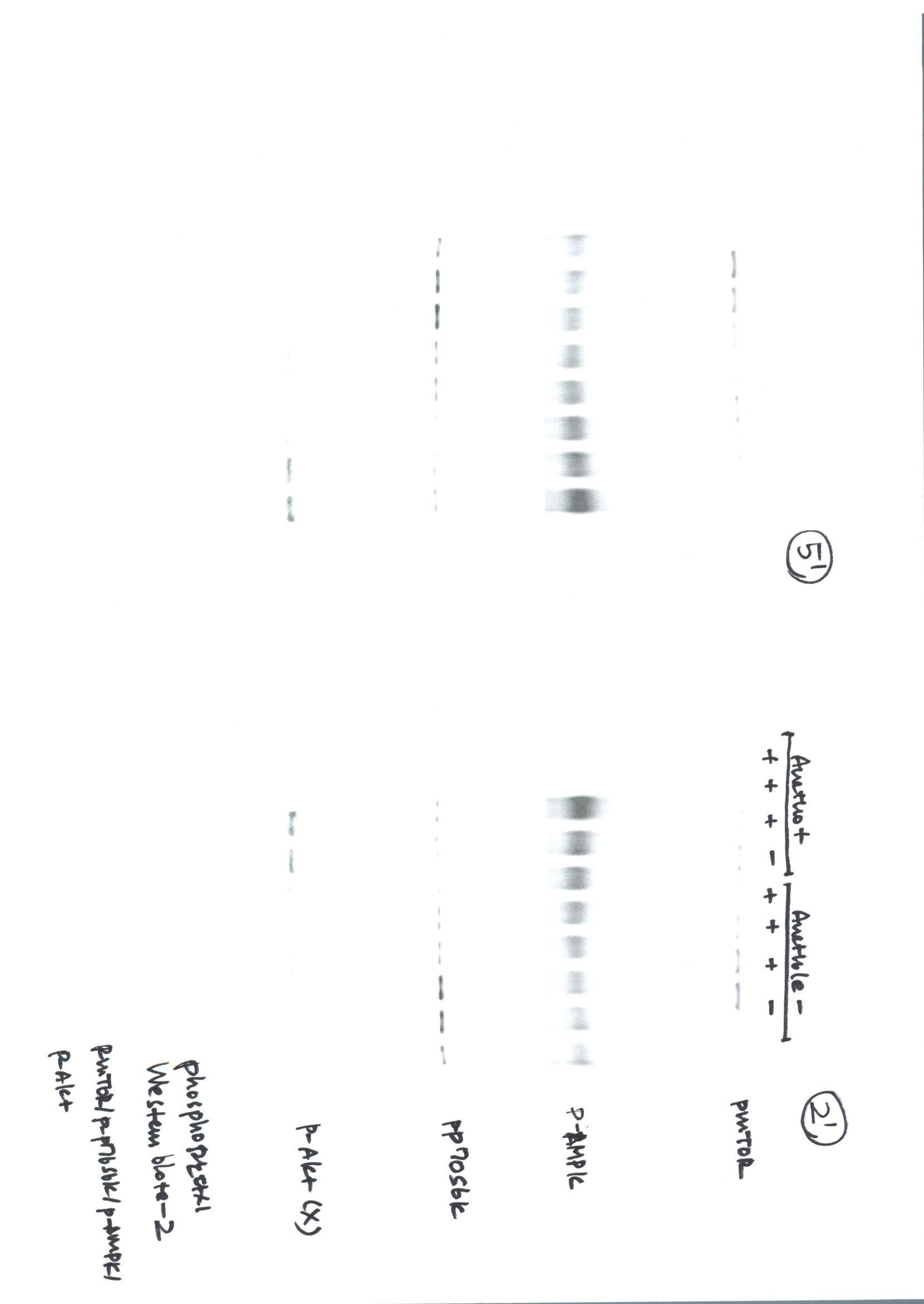

Supplement: Supplementary file 3 — The raw data of western blot. p-mTOR, p-AMPK, and p-p70S6K in Fig. 2. (JPG 114 kb) [file 12860_2018_163_MOESM3_ESM.jpg]

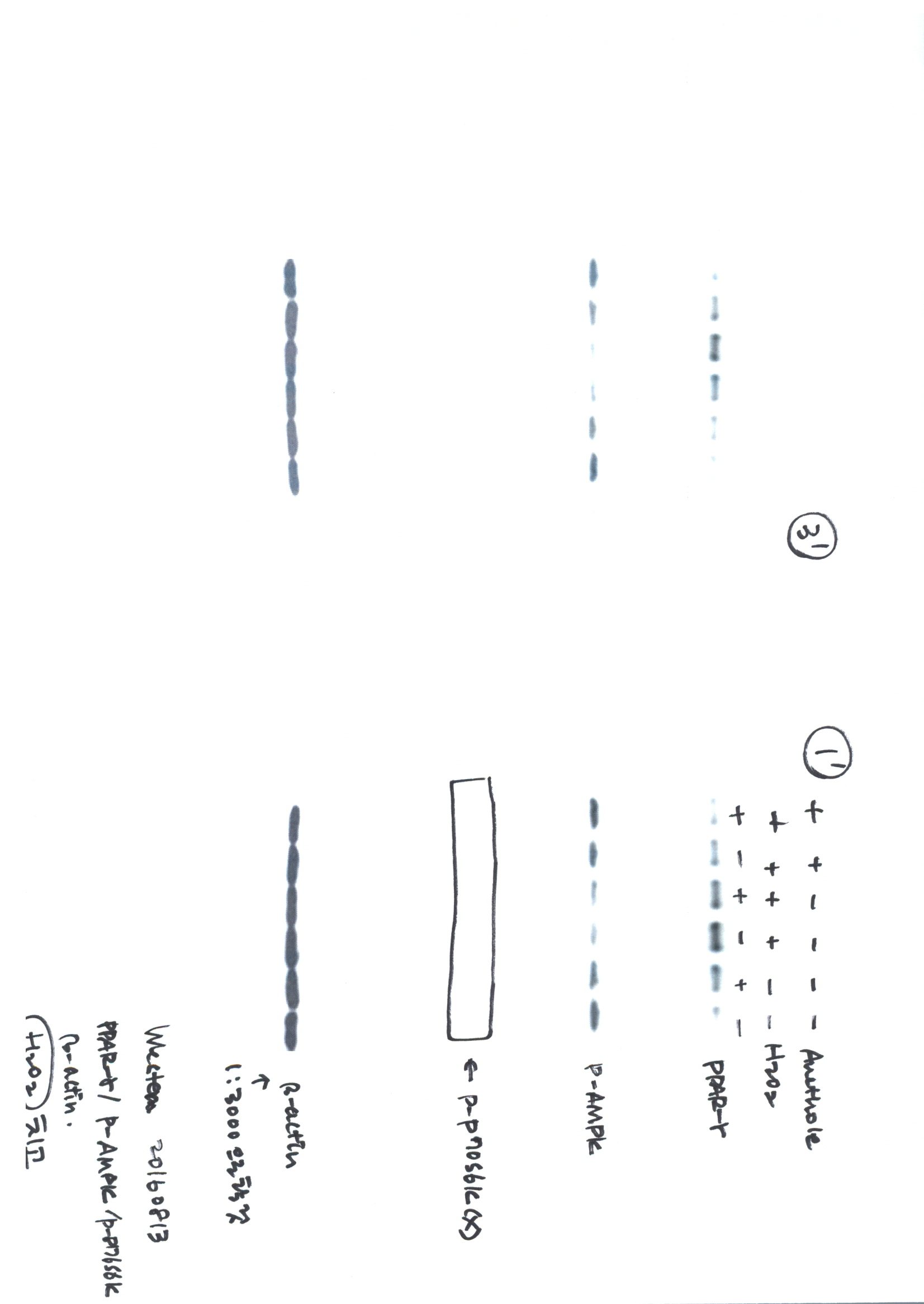

Supplement: Supplementary file 4 — The raw data of western blot. PPAR-γ, p-AMPK, and and β-actin in Fig. 4. (JPG 120 kb) [file 12860_2018_163_MOESM4_ESM.jpg]

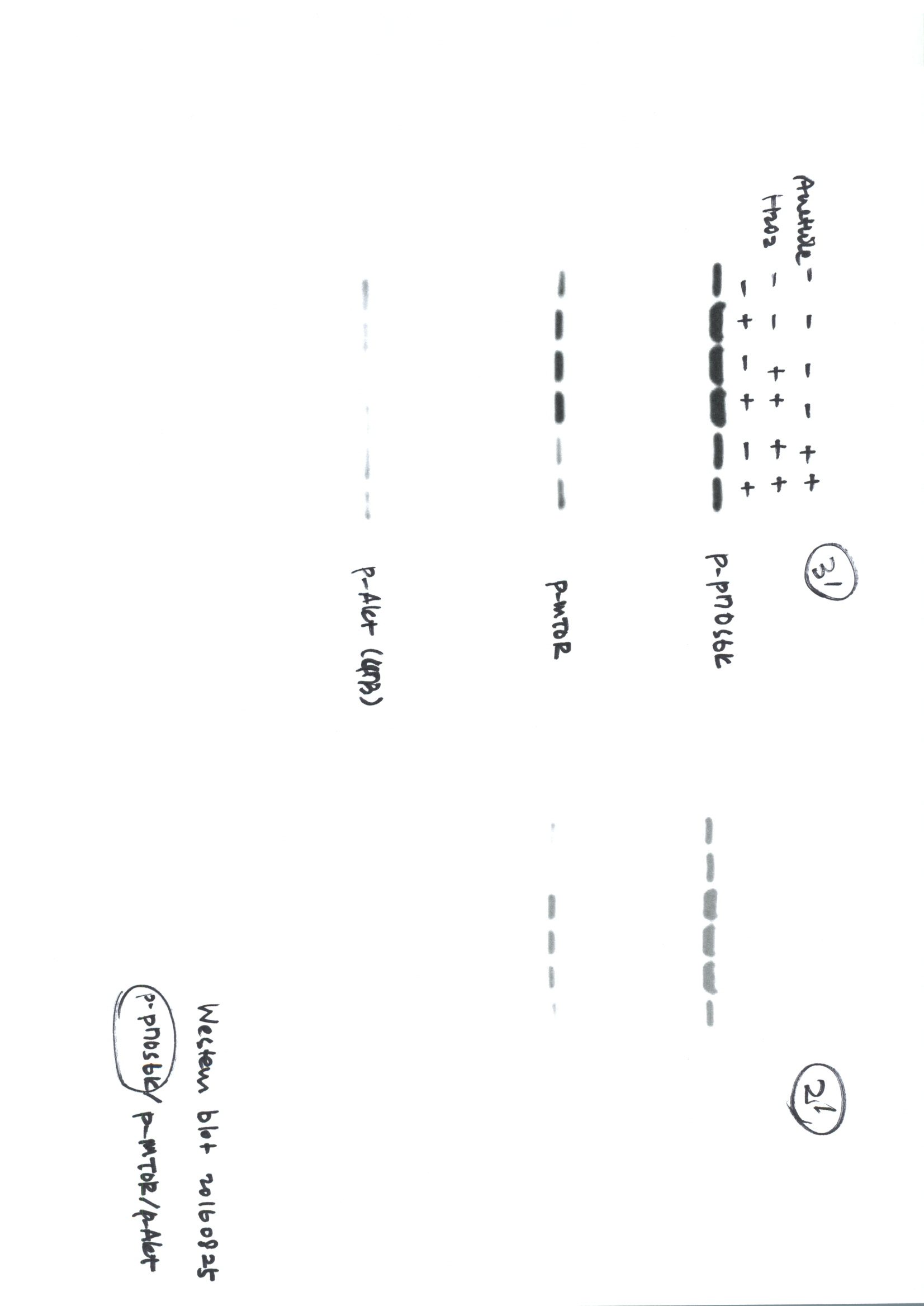

Supplement: Supplementary file 5 — The raw data of western blot. p70S6K, p-mTOR, and p-Akt in Fig. 4. (JPG 106 kb) [file 12860_2018_163_MOESM5_ESM.jpg]
